# Vascular Senescence in Cardiovascular and Metabolic Diseases

**DOI:** 10.3389/fcvm.2018.00018

**Published:** 2018-03-05

**Authors:** Goro Katsuumi, Ippei Shimizu, Yohko Yoshida, Tohru Minamino

**Affiliations:** ^1^Department of Cardiovascular Biology and Medicine, Niigata University Graduate School of Medical and Dental Sciences, Niigata, Japan; ^2^Division of Molecular Aging and Cell Biology, Niigata University Graduate School of Medical and Dental Sciences, Niigata, Japan

**Keywords:** cellular senescence, p53, atherosclerosis, heart failure, diabetes, senolysis

## Abstract

In mammals, aging is associated with accumulation of senescent cells. Stresses such as telomere shortening and reactive oxygen species induce “cellular senescence”, which is characterized by growth arrest and alteration of the gene expression profile. Chronological aging is associated with development of age-related diseases, including heart failure, diabetes, and atherosclerotic disease, and studies have shown that accumulation of senescent cells has a causative role in the pathology of these age-related disorders. Endothelial cell senescence has been reported to develop in heart failure and promotes pathologic changes in the failing heart. Senescent endothelial cells and vascular smooth muscle cells are found in atherosclerotic plaque, and studies indicate that these cells are involved in progression of plaque. Diabetes is also linked to accumulation of senescent vascular endothelial cells, while endothelial cell senescence *per se* induces systemic glucose intolerance by inhibiting skeletal muscle metabolism. A close connection between derangement of systemic metabolism and cellular senescence is also well recognized. Aging is a complex phenomenon, and there is no simple approach to understanding the whole process. However, there is accumulating evidence that cellular senescence has a central role in the development and progression of various undesirable aspects of aging. Suppression of cellular senescence or elimination of senescent cells reverses phenotypic changes of aging in several models, and proof-of-concept has been established that inhibiting accumulation of senescent cells could become a next generation therapy for age-related disorders. It is clear that cellular senescence drives various pathological changes associated with aging. Accordingly, further investigation into the role of this biological process in age-related disorders and discovery of senolytic compounds are important fields for future exploration.

## Introduction

In aging societies, the discrepancy between the total lifespan and the healthy lifespan is becoming a major problem. Chronological aging is associated with a higher prevalence of age-related diseases, including heart failure, diabetes, and atherosclerotic disorders with or without various comorbidities, resulting in impairment of the quality of life by limitation of normal activities. Thus, aging is associated with several undesirable processes. The mechanisms of aging and age-associated disorders are complex, and thus cannot be comprehended by a simple approach. However, recent studies have indicated a pivotal role of cellular senescence in the progression of age-related disorders (1–5). Back in the 1960s, Hayflick et al. demonstrated that fibroblasts have limited potential to replicate (6), indicating that aging also occurs at the cellular level. Such aging of cells is currently described as “cellular senescence”. Senescent cells become enlarged and flattened. In association with proliferative arrest, alterations of gene expression by these cells lead to secretion of pro-inflammatory molecules (7). This is known as the senescence-associated secretory phenotype, and it results in chronic sterile inflammation that promotes tissue remodeling. Senescent cells have been found in various organs of animal models, as well as in elderly humans and persons with age-related disorders. There is evidence that cellular senescence in the vasculature, termed “vascular senescence”, is crucially involved in the pathogenesis of cardiovascular and metabolic disorders. Vascular senescence has been reported to promote atherosclerosis (8), systolic cardiac dysfunction (9, 10), and systemic metabolic dysfunction (11). In this review, we delineate the role of cellular senescence in the diseases associated with aging, focusing on vascular senescence in cardiovascular and metabolic disorders, and discuss the potential usefulness of therapies targeting senescent cells.

### Role of Cellular Senescence in Aging and Age-Related Diseases

**Figure 1:**
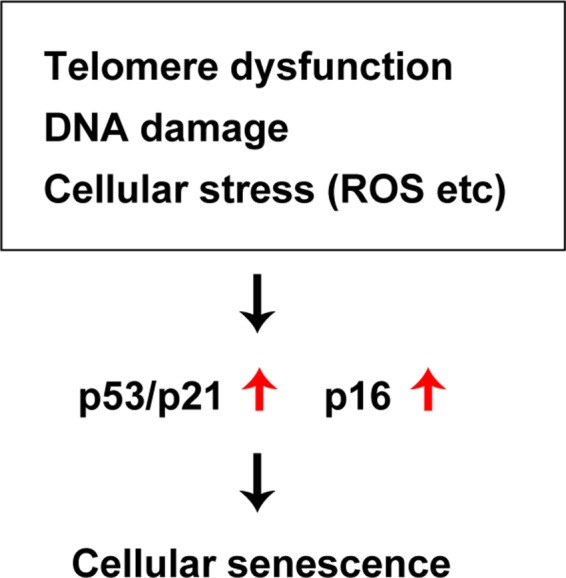
Common pathways of cellular senescence. Telomere dysfunction, DNA damage, cellular stress (ROS etc) up-regutate p53/p21, p16 signal and induces cellular senescence.

Diverse deleterious changes of cells and tissues accumulate with the progression of aging, leading to a decline of physiological activity and an increased risk of death. Organ function deteriorates with chronological aging, and this biological process is characterized by cellular senescence, changes of intercellular communication, mitochondrial dysfunction, and deregulation of nutrient sensing (1). It is well known that DNA damage, telomere shortening, oncogenic stress, and exposure to high levels of reactive oxygen species (ROS) all occur with chronological aging, and signaling via the p53 pathway has been reported to increase under these conditions (12). The p53 protein is a transcriptional factor with a crucial role in maintenance of genomic stability that mediates the coordination of DNA repair, cell cycle regulation, apoptosis, and cellular senescence. Because p53 is involved in suppression of tumorigenesis, it has been described as the “guardian of the genome” (13). In addition to its role in the repair of DNA damage and maintenance of genomic stability, studies have indicated that p53 contributes to a broad spectrum of biological processes, such as cell metabolism, autophagy, antioxidant defenses, and angiogenesis (13–15). Accordingly, p53 signaling is thought to have a central role in cellular senescence. Somatic cells have a finite lifespan and eventually enter a state of irreversible growth arrest termed “replicative senescence.” Telomeres are repetitive nucleotide sequences located at the terminals of mammalian chromosomes that undergo incomplete replication during cell division, resulting in telomere shortening. Because telomeres are essential for chromosomal stability and DNA replication, DNA damage is recognized when telomere shortening exceeds the physiological range and this triggers cellular senescence, mainly via the p53 or p16 signaling pathways. “Stress-induced premature senescence” is another type of cellular senescence that is triggered by various stress signals, including DNA damage induced by oxidative stress or irradiation, constitutive activation of mitogenic stimuli, oncogenic activation, and metabolic stress. It is also mediated via the p53 or p16 signaling pathways. Preference for one pathway over the other depends on the cell type and also varies among species (16, 17). In humans, telomere dysfunction activates either p53 or p16 signaling, while only p53 signaling is activated in rodents (18). It is generally accepted that p53 signaling is primarily activated by DNA damage and telomere dysfunction, while p16 signaling is primarily linked to mitogenic stress, chromatin disruption, and general cellular stress (16, 17, 19) (Figure 1).

It was reported that p53 is increased in the failing heart, in aged vessels, and in the visceral fat of patients with obesity or heart failure. Studies have indicated a pathological role of p53-induced cellular senescence in aging and age-related disorders, including heart failure, atherosclerotic disease, obesity, and diabetes (10, 11, 15, 20–24). However, there is controversy about the role of p53 in aging and age-related diseases (3, 25, 26). In some settings, p53 signaling has been shown to have a beneficial effect by suppression of aging. Matheau and colleagues reported that Trp53/Cdkn2a transgenic mice were resistant to carcinogenesis and had a longer median lifespan (27). It was also reported that Trp53 transgenic (“Super p53”) mice displayed resistance to carcinogenesis without any signs of premature aging, and these mice showed normal glucose tolerance on a standard diet (28–30). Furthermore, Baker et al. found that loss of p53 or p21 accelerated cellular senescence in the adipose tissue and skeletal muscle of BubR1 progeroid mice (31). They also reported that p19^Arf^, acting upstream of p53, suppressed senescence and aging in the same progeroid model (32). Taken together, these various reports suggest that the p53/p21 signaling pathways regulate cellular senescence in a context-dependent manner.

Interestingly, it was recently reported that elimination of senescent cells by genetic manipulation inhibited age-related degenerative changes in several organs of mice, such as the heart and kidneys (33). Other studies have identified several pharmacological agents that selectively damage and remove senescent cells, and these compounds have been described as “senolytic agents”. For example, an inhibitor of anti-apoptotic proteins (ABT263) depletes senescent bone marrow hematopoietic stem cells and senescent muscle cells in a chronological aging model, leading to rejuvenation of these tissues (34). The gene expression profile of senescent cells is shifted toward a pro-inflammatory phenotype associated with the secretion of biologically active molecules (senescence-associated secretory phenotype). *In vitro* studies have shown that exposure of young fibroblasts to senescent fibroblast promotes senescence of the young cells via a gap junction-mediated process, which has been described as the “bystander effect” (35). Studies have shown that senescent cells damage their local environment and promote tissue remodeling in age-related disorders, suggesting that inhibition of cellular senescence and/or elimination of senescent cells could be potential next generation therapies for diseases associated with aging.

### Biological Markers of Cellular Senescence

Biological markers reflecting direct evidence of cellular senescence have not yet been identified, but several markers are used to indirectly detect senescent cells, among which senescence-associated beta-galactosidase (SA-β-gal) activity is the most common. Lysosomal beta-galactosidase activity is normally detected at a low pH (usually around pH 4), but becomes detectable at a higher pH (pH 6) in senescent cells due to marked expansion of the lysosomal compartment (36). Other established markers of cellular senescence include high expression of p53, p16, p21, p38 mitogen-activated protein kinase (p38MAPK) and γH2AX, reflecting the activation of DNA damage responses (4, 37–40). In addition, high mobility group A (HMGA) proteins or heterochromatin markers, including HP1 and tri-methylated lysine 9 histone H3 (H3K9me3), are recognized as molecular markers of senescence-associated heterochromatin foci and are considered to indicate cellular senescence (40).

### Cardiac Aging Predisposes to Heart Failure

Heart failure has a high prevalence among the elderly (41). The prognosis of severe heart failure is still unacceptably poor, and there is an urgent need to find better therapies for this condition. Age-related heart failure develops in persons without established risk factors, such as hypertension, obesity, diabetes, or atherosclerotic diseases (42, 43). Heart failure without systolic dysfunction is classified as heart failure with a preserved ejection fraction (HFpEF), and occurs in approximately half of all patients with heart failure. HFpEF is prevalent among the elderly and lack of specific therapy for this type of heart failure is a major clinical problem. The mechanism of HFpEF is still not fully understood, although there is evidence of cardiac endothelial cell remodeling being involved in its onset and progression (44). It was also reported that coronary microvascular endothelial inflammation is critically involved in the pathology of HFpEF (45), while a recent study indicated a causative role of senescent signaling in this disorder (46). Thus, the physiological aging process seems to increase susceptibility to the onset of heart failure, considering that the prevalence of heart failure increases with age. Various studies have indicated that cellular senescence is critically involved in the pathology of heart failure, as described below.

### Vascular Senescence and Heart Failure

#### Endothelial Cell Senescence

**Figure 2:**
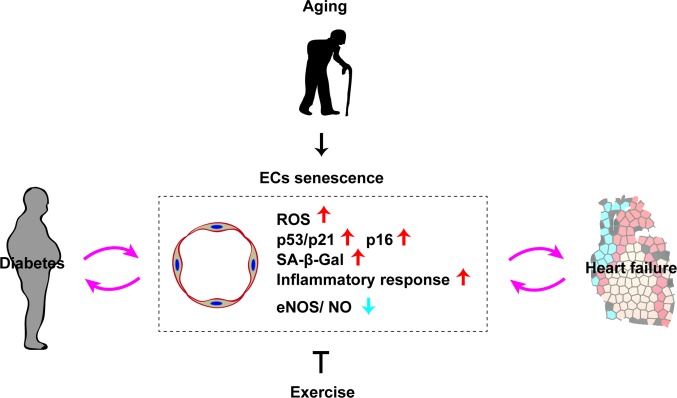
Role of endothelial senescence in cardio-metabolic disease. Chronological aging induces vascular endothelial cell senescence (EC senescence as characterized with an increase of ROS, p53/p21, p16, SA-β-Gal, inflammatory response, and reduced eNOS/NO level). EC senescence has a pivotal role in the progression of diabetes and heart failure. Exercise has a potential to suppress ECs senescence.

Although the role of cellular senescence in the failing heart is still not fully understood, a number of studies have suggested a pathological influence on heart failure. The cardiac level of p53 is increased in a murine model of left ventricular pressure overload, leading to suppression of myocardial angiogenesis that results in capillary rarefaction, tissue hypoxia, and cardiac dysfunction (15). Chronic sterile inflammation develops in the failing heart, and it is now well accepted that such inflammation is one of the mechanisms underlying cardiac remodeling (47). It was recently demonstrated that activation of p53 signaling in vascular endothelial cells induces cardiac inflammation and remodeling in a murine model of left ventricular (LV) pressure overload (10). Expression of p53 by capillary endothelial cells in the left ventricle increases in response to LV pressure overload, leading to elevated expression of intercellular adhesion molecule (ICAM)−1 by these cells that promotes infiltration of macrophages and cardiac inflammation. Conversely, depletion of p53 from capillary endothelial cells results in suppression of ICAM-1 expression and cardiac inflammation with improvement of cardiac dysfunction. Activation of the sympathetic nervous system occurs in heart failure and is associated with a poor prognosis (48). It was reported that the sympathetic nervous system/ROS axis increases p53 expression by endothelial cells in a murine model of LV pressure overload (10). In another study, depletion of p53 from endothelial cells improved capillary rarefaction and cardiac function, while suppressing cardiac fibrosis and remodeling (9). These findings indicate that endothelial p53 signaling suppresses angiogenesis, thereby promoting capillary rarefaction in the failing heart. Inhibition of p53 in endothelial cells could potentially become a next generation therapy for patients with heart failure and a reduced ejection fraction. As mentioned above, about half of all heart failure patients have HFpEF with a preserved ejection fraction. There are several established risk factors for HFpEF, including overweight/obesity, hypertension, diabetes, and aging. Cardiomyocyte hypertrophy and interstitial fibrosis develop in patients with HFpEF, leading to incomplete myocardial relaxation and increased wall stiffness (49–51). It is generally accepted that coronary microvascular inflammation is central to the pathogenesis of HFpEF (45), and it was recently demonstrated that endothelial cell senescence also makes a contribution. When mice with accelerated senescence were fed a high-fat, high-salt diet, both endothelial cell senescence and inflammation increased in cardiac tissue, along with the typical hemodynamic and structural changes of HFpEF (46). Considering that cellular senescence induces vascular dysfunction and inflammation, it seems reasonable that it would also promote pathologic changes of HFpEF. Accordingly, suppression of endothelial cell senescence may be a therapeutic option for this currently untreatable disorder (Figure 2).

## Vascular Aging Predisposes to Atherosclerotic Diseases

### Structural and Functional Changes of Aging Arteries

Coronary artery disease and stroke are associated with arterial dysfunction. Arterial remodeling occurs with aging, even in the absence of cardiovascular disease and cardiovascular risk factors. While aging is a physiological process and not a pathological condition, studies indicate that aging *per se* is linked with vascular remodeling that predisposes to cardiovascular disease. Aged arteries are characterized by an increase of the intima/media thickness ratio, which was reported to increase by 2- to 3-fold from 20 to 90 years of age (52, 53). Vascular smooth muscle cells switch from the “contractile” to “synthetic” phenotype with aging and this change contributes to intimal thickening, which is associated with increased arterial permeability and leads to development of atherosclerotic disease. The arterial media also becomes thicker with aging and its cellularity decreases simultaneously (54). Moreover, the length and circumference of the aorta increase with aging (55), and these structural changes reflect increased collagen production and a corresponding decline of the elastin content (56). In association with such changes, aged vessels show reduced compliance, reduced elasticity/distensibility, and increased stiffness, resulting in a higher systolic blood pressure and lower diastolic pressure (57). Medial calcification is another characteristic of aged vessels. In association with other age-related disorders like hypertension, dyslipidemia, and diabetes, such vascular remodeling increases susceptibility to atherosclerotic vascular diseases. In elderly patients, atherosclerotic plaques tend to become larger and vascular stenosis becomes more severe over time. Aged rabbits fed a high fat diet developed more severe atherosclerotic lesions compared to young animals on the same diet. Therefore, it is well accepted that aging *per se* promotes the pathogenesis of atherosclerotic disorders, and studies have suggested that cellular senescence has a critical role in this process.

### Vascular Senescence in Arterial Diseases

**Figure 3:**
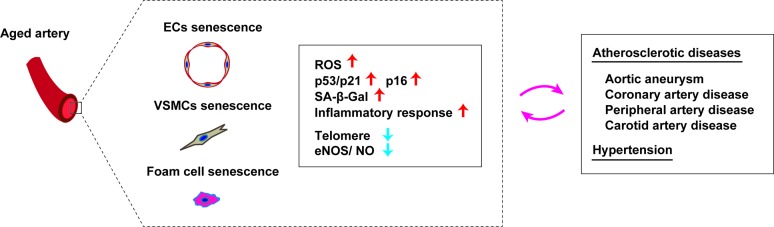
Cellular senescence in aged arteries. Aged arteries are characterized by accumulation of senescent vascular endothelial cells (EC), senescent vascular smooth muscle cells (VSMCs), and senescent foam cells. Accumulation of these senescent cells are associated with an increase of ROS, p53/p21, p16, SA-β-Gal, inflammatory response, telomere attrition and reduced eNOS/NO level). Senescence of these cells promotes pathological changes in atherosclerotic diseases and also has a role in development of hypertension.

ROS and chronic low-grade sterile inflammation are two major contributors to the progression of age-related vascular dysfunction. Senescent cells accumulate in the arteries with aging irrespective of whether or not a person has age-related vascular disorders (58–61). Along with aging, vascular tissues of rodents and humans show elevation of the levels of p16, p21, phosphorylated p38, and double-stranded DNA breaks, in association with high SA-β Gal activity (62–65). It was reported that expression of p53 and p21 is increased in the arteries of elderly persons, together with structural breakdown of telomeres known as telomere uncapping (61). Both telomere length and telomerase activity were found to be reduced in endothelial progenitor cells from patients with coronary heart disease (66). In patients with chronic heart failure, telomere attrition was identified in circulating leukocytes (67). Moreover, the leucocyte telomere length displays an inverse association with the risk of coronary heart disease independently of conventional vascular risk factors (68). Accordingly, it is generally accepted that telomere length and telomerase activity are involved in human cardiovascular disease (69). Interestingly, senescent cells are increased in the coronary arteries of patients with ischemic heart disease, but not in the internal mammary arteries (58). Endothelial cells and vascular smooth muscle cells (VSMCs) from patients with abdominal aortic aneurysm (AAA) have the phenotypic features commonly observed in senescent cells (60). Hypertension is an established risk factor for atherosclerotic diseases, and it was reported that binding of p53 to the p21 promoter is increased in the arteries of hypertensive patients. While telomere length is comparable between patients with hypertension and controls, telomere uncapping is 2-fold higher in hypertensive patients (70). A murine model of genomic instability demonstrated senescence of endothelial cells and VSMCs in the aorta, along with impaired vasodilation, increased vascular stiffness, and hypertension (71). In hypertensive rats treated with deoxycorticosterone acetate and salt, overexpression of p16 was detected in the coronary arteries (72). Aortic p16 expression was elevated in another model of hypertension (mice administered an endothelial nitric oxide synthase inhibitor) (73), indicating the existence of a vicious circle between cellular senescence and hypertension. Thus, studies have shown that senescent cells accumulate in the vessels of patients with atherosclerosis, hypertension, aneurysms, diabetes, and intimal hyperplasia (Figure 3), so the role of endothelial cell, VSMC and immune cell senescence in arterial diseases is discussed next.

### Endothelial Cell Senescence in Arterial Diseases

Endothelial cells are critically important for maintaining vascular homeostasis and are involved in various biological functions, including angiogenesis, blood pressure regulation, coagulation, and systemic metabolism. Aged endothelial cells develop a dysfunctional phenotype that is characterized by reduced proliferation and migration, decreased expression of angiogenic molecules, and low production of nitric oxide (NO), which is synthetized by NO synthase (NOS) and mediates vasodilatation. In dysfunctional endothelial cells, NO production is generally reduced due to low NOS activity. This change is associated with impairment of endothelium-dependent dilatation (EDD), which is reported to predict future cardiovascular events. Dysfunctional endothelial cells develop pro-oxidant, pro-inflammatory, vasoconstrictor, and prothrombotic properties, and studies indicate that cellular senescence has a pathological role in such phenotypic aging.

Senescent endothelial cells have been found in atherosclerotic plaque (58). An autopsy study of patients with ischemic heart disease revealed that SA-β-gal activity is increased in the coronary arteries, but not in the internal mammary arteries. In the coronary arteries, SA-β-gal activity is high in cells located on the luminal surface (probably endothelial cells). ICAM-1 is also increased in senescent human aortic endothelial cells, while both endothelial nitric oxide synthase (eNOS) and NO activity are reduced in these cells compared to young cells. Importantly, these pathological phenotypic changes induced by replicative senescence were suppressed by activation of telomerase reverse transcriptase in aged human aortic endothelial cells, indicating that telomere shortening induces endothelial cell senescence and has pathological consequences in atherosclerotic diseases (58). In patients with AAA, telomeres are significantly shorter and oxidative DNA damage is more severe in endothelial cells from the aneurysmal region (60). In atherosclerotic mice, disturbance of flow in the ascending aorta and aortic arch promotes endothelial cell senescence, and *in vitro* studies have indicated that aberrant flow is a signal inducing cellular senescence (74). One of the problems related to an increase of senescent cells is development of the senescence-associated secretory phenotype, which is characterized by production of pro-inflammatory cytokines with a causal role in tissue remodeling (7). In human arterial endothelial cells with replicative senescence, levels of H_2_O_2_ and O_2_^–^ are high and NO production is reduced. High ROS levels in senescent endothelial cells are thought to accelerate senescence. Aging is reported to be linked with increased circulating levels of pro-inflammatory cytokines, such as interleukin-6, tumor necrosis factor alpha, and monocyte chemoattractant protein-1 (75). It is highly possible that accumulation of senescent endothelial cells in the arteries of elderly persons induces chronic sterile inflammation and vascular remodeling, increasing susceptibility to atherosclerotic diseases. Controversy exists as to whether physical activity is associated with telomere length, since physical activity is positively correlated with telomere length in some studies, but not in other studies (76). It is generally accepted that physical activity improves vascular structure and function in humans and rodents. Bioavailability of NO declines with aging in association with elevation of ROS levels, while these changes are ameliorated or reversed by physical activity. Recently, it was shown that older individuals who performed exercise had lower levels of p53, p21, and p16 in endothelial cells from the brachial arteries and antecubital veins compared to sedentary older individuals, indicating that physical activity suppresses senescence of human vascular cells (77). Thus, there is evidence that physical activity improves vascular function, but further studies are needed to identify the detailed molecular mechanisms involved (Figure 2).

### Vascular Smooth Muscle Cell Senescence in Arterial Diseases

Functional changes of VSMCs occur with aging, partly due to deregulation of TGF-β signaling, and these cells undergo transformation from a “contractile” to a “synthetic” phenotype. It was reported that aged SMCs show enhancement of inducible NOS (iNOS) activity, as well as higher expression of ICAM-1 and angiotensinogen in response to stress (78, 79). Intimal thickening develops with aging, partly due to increased production of collagen and a corresponding decrease of elastin (56). Generally, intimal thickening is widespread and concentric in the aorta, while it is eccentric in the coronary arteries. Intimal thickening is a preatherosclerotic lesion because topographic correspondence has been demonstrated between the sites of intimal thickening and atherosclerotic plaques. Telomeres are shorter in cells from atherosclerotic plaque in the human aorta compared to normal vessels. Telomeres are also shorter in VSMCs from the fibrous cap of atheroma compared to VSMCs from the normal vascular media, and these cells are positive for SA-β-gal staining along with elevated p16 and p21 expression. It was reported that oxidative stress induces DNA damage in VSMCs and suppresses telomerase activity, leading to telomere shortening and cellular senescence that contribute to acceleration of atherosclerotic disorders (80). In addition, senescence of VSMCs was reported in apolipoprotein E null mice treated with angiotensin II (81). Smooth muscle 22α is an actin-binding protein that is known as a marker of smooth muscle cell senescence. Recently, it was shown that smooth muscle 22α promotes angiotensin II-induced cellular senescence by suppressing Mdm-2-mediated degradation of p53 (82). In addition, Cafueri et al. reported that VSMCs from the aneurysms of AAA patients display telomere attrition and marked oxidative DNA damage (60). Senescent VSMCs have been identified in atherosclerotic lesions of patients with coronary artery disease, AAA, and peripheral artery disease (58). It was also reported that 18% of VSMCs from carotid artery plaques are positive for p16 and p21, in association with detectable SA-β-gal activity (80). Furthermore, SA-β-gal positive VSMCs in carotid plaques express interleukin-6, suggesting that senescent VSMCs have a senescence-associated secretory profile and a causative role in the progression of atherosclerotic disorders (83). Senescent VSMCs show dysregulated production of pro-inflammatory cytokines, growth factors, and extracellular matrix modifiers, accelerating the process of vascular remodeling. Some inducers of senescence are common to VSMCs and vascular endothelial cells, such as ROS and angiotensin II (84, 85), while hypoxic stress was reported to inhibit senescence by promoting telomerase activity (86). It was recently found that senescent VSMCs in atherosclerotic plaque display loss of telomeric repeat-binding factor-2 (TRF2), a protein localized in the telomeres. TRF2 overexpression reduces DNA damage, accelerates DNA repair, and suppresses cellular senescence *in vitro*, while introduction of TRF2 into loss of function mutants results in the opposite phenotype. Studies using transgenic mice have shown that VSMC-specific loss of TRF2 function increases atherosclerosis and necrotic core formation *in vivo*, while these pathological changes are suppressed in mice with VSMC-specific gain of TRF2 function (87). These results clearly indicate that inhibition of VSMC senescence is extremely important for suppressing the progression of atherosclerotic diseases.

### Immune Cell Senescence in Arterial Diseases

In human vascular cells, telomere length shows a strong inverse correlation with aging, and telomere shortening is linked with cardiovascular disease and diabetes (88, 89). Elderly individuals with short telomeres in leukocyte DNA are reported to have a higher mortality rate, partly attributable to increased death from heart disease (90). Monocytes from patients with atherosclerosis exhibit increased production of ROS and various pro-inflammatory cytokines, including MCP-1, IL-6, IL-1*β,* and TNF-*α* (91). It was recently demonstrated that senescent intimal foam cells accumulate in atherosclerotic lesions and act as the key drivers of atheroma formation. Importantly, specific deletion of these senescent cells by genetic or pharmacological approaches has been shown to reverse atherosclerosis in mice (92). According to another report, cellular senescence mediated by p16^INK4a^ promotes pro-inflammatory phenotypic changes in macrophages (93). These studies indicate that leukocyte senescence is involved in the progression of atherosclerotic plaque, suggesting that suppression of leukocyte senescence may be an important approach for combating atherosclerotic diseases.

### Vascular Senescence in Metabolic Syndrome

Chronic sterile inflammation of visceral fat develops in patients with heart failure, obesity, and/or diabetes, and is well accepted to have a central role in inducing systemic insulin resistance and progression of metabolic disorders. It was previously reported that p53 induces inflammation of visceral adipose tissue in murine models of obesity or heart failure, which is involved in the progression of these age-related diseases (21, 24, 94). Occurrence of cellular senescence in visceral fat was reported to result in deterioration of systemic metabolic health. Capillaries have a crucial role in metabolically active organs, including skeletal muscle and brown adipose tissue. Capillarization of skeletal muscle was reported to increase in older adults after exercise training, and this increase of capillaries leads to enhancement of systemic insulin sensitivity (95). It was recently demonstrated that vascular endothelial cell senescence induces systemic metabolic dysfunction (11) (Figure 2). Conversely, it has been well documented that obesity is associated with vascular senescence. In metabolically unhealthy persons with obesity and/or diabetes, insulin/insulin receptor/insulin receptor substrate/phosphoinositide 3-kinase/Akt signaling is down-regulated in vascular cells, while insulin receptor/son of sevenless/growth factor receptor bound protein/mitogen-activated protein kinase (MAPK) signaling is enhanced. This is known as “selective insulin resistance”, and it mediates pro-atherosclerotic responses by activation of MAPK signaling. It was reported that activation of MAPK signaling by hyperinsulinemia and selective insulin resistance induces vascular remodeling through vasoconstriction, proliferation, and vascular cell migration (96). Various studies have indicated the existence of a vicious circle between vascular senescence and metabolic syndrome, as discussed below.

### Endothelial Cell Senescence in Metabolic Syndrome

Capillary network formation is critically important for morphogenesis and maintenance of homeostasis, while vascular dysfunction induces organ malfunction and systemic metabolic disorders (97, 98). In animal studies, diabetes has been shown to induce vascular cell senescence. For example, endothelial cell senescence develops in the aortas of Zucker diabetic rats and hyperglycemic mice or rats (99–101). These reports indicate that hyperglycemia induces cellular senescence, while there is also evidence that cellular senescence *per se* promotes systemic metabolic dysfunction. It is widely accepted that skeletal muscle contributes to glucose disposal, so maintaining skeletal muscle homeostasis is crucial for systemic metabolic health. Metabolic stress induces accumulation of lipids and chronic sterile inflammation in skeletal muscle, contributing to development of systemic insulin resistance (102, 103). It was recently demonstrated that endothelial cell senescence suppresses skeletal muscle metabolism, leading to systemic glucose intolerance. Metabolic stress induced by dietary obesity increases p53 expression in the vascular endothelium (11), while endothelial cell-specific depletion of p53 reduces both visceral and subcutaneous fat volumes and improves systemic glucose intolerance. It is generally accepted that eNOS has a protective role in the cardiovascular system, which is mainly mediated by production of NO. It was reported that eNOS up-regulates skeletal muscle expression of peroxisome proliferator-activated receptor-γ coactivator-1α, a master regulator of mitochondrial biogenesis and cell metabolism, while this up-regulation is suppressed by p53. Down-regulation of p53 expression in vascular endothelial cells promotes glucose uptake by skeletal muscle through up-regulation of glucose transporter-1 expression in endothelial cells, and contributes to better systemic metabolic health. These findings indicate that suppression of endothelial cell senescence is important for maintenance of systemic metabolic health (11).

### Potential Next Generation Therapies Targeting Senescent Cells for Cardiovascular and Metabolic Disorders

It was recently established that selective depletion of senescent cells (“senolysis”) reverses phenotypic changes of aging (33, 34, 104–106). Accumulation of senescent cells promotes chronic sterile inflammation in the visceral adipose tissue of patients with obesity and elderly persons. In a murine model of premature aging, elimination of p16-positive senescent cells contributed to suppression of the aging phenotype in several organs, including epididymal/inguinal white adipose tissue, the heart, and the kidney (33). Several agents causing selective depletion of senescent cells (senolytic activity) have been identified. It was reported that an anticancer agent (ABT263) has a senolytic effect by selectively removing p16-positive senescent cells from the bone marrow via apoptosis, leading to rejuvenation of hematopoietic stem cells during aging (34). In addition, Xu et al. showed that depletion of senescent cells in aged mice preserved adipogenesis and increased insulin sensitivity (104). Zhu et al. showed that administration of another senolytic therapy (dasatinib +quercetin: D + Q) significantly improved systolic cardiac function and reduced the left ventricular end-systolic dimension in 24-month-old mice (105). Treatment with D + Q also improved vasomotor function in aged mice, as well as reducing aortic calcification and osteogenic signaling in hypercholesterolemic mice (107). Regarding genetic approaches, elimination of p16-positive senescent cells from plaques suppressed pathologic changes in low-density lipoprotein receptor-deficient mice (92). Senescent VSMCs show hypermetabolic changes, with increased glycolysis and oxygen consumption. Administration of 2-deoxyglucose causes greater depletion of senescent VSMCs than control VSMCs *in vitro*, but clinical trials investigating the anticancer effect of 2-deoxyglucose have identified the issue of toxicity (108). Depletion of specific components to alter cell metabolism has now attracted attention in the field of anticancer therapy, including the search for senolytic agents targeting cell metabolism. There is evidence that elimination of senescent cells by administration of senolytic agents has the potential to become a next generation therapy for cardiovascular disorders (109, 110). Suppression of cellular senescence is another possibility. Sirtuin1 (SIRT1) is one of the most promising molecules to be studied in relation to suppression of aging. Activation of SIRT1-signaling was reported to prolong the lifespan of rodents, while overexpression of SIRT1 in VSMCs or vascular endothelial cells suppresses senescence and extends the survival of these cells. Resveratrol activates SIRT1, and administration of resveratrol was reported to prevent arterial wall inflammation and elevation of the pulse wave velocity by dietary obesity (111). Moreover, activation of SIRT1 attenuates arterial stiffness and hypertension in Klotho-haplodeficient mice (112). SIRT1 expression and activity are decreased in the VSMCs of patients with AAA, together with vascular cell senescence and elevated p21 expression, while SIRT1 inhibits p21-induced cellular senescence and contributes to suppression of vascular inflammation (113). Another study showed that calorie restriction up-regulates SIRT1 expression in vascular smooth muscle cells, and reduced the incidence of AAA (114). These results indicate that activation of SIRT1 in VSMCs may potentially prevent the progression of AAA (113, 114). Nicotinamide adenine dinucleotide (NAD) is a coenzyme involved in cell metabolism, redox reactions, and DNA repair, and it is well known to suppress aging (115). Nicotinamide phosphoribosyltransferase (Nampt) is the rate-limiting enzyme for conversion of nicotinamide to nicotinamide mononucleotide, enabling subsequent biosynthesis of NAD^+^. Nampt overexpression was reported to suppress senescence of VSMCs through a process mediated by SIRT1 signaling (116). In patients with aortic dilation, an inverse relationship between Nampt expression by VSMCs and the diameter of the ascending aorta was recently identified, and the authors concluded that NAD^+^ biosynthesis in the aortic media is important for protection against DNA damage and premature VSMC senescence (117). Systemic metabolic dysfunction is reported to induce cellular senescence in endothelial cells. Aged mice with systemic glucose intolerance and hyperinsulinemia show elevation of aortic NADPH oxidase-2 (Nox2) expression, while *in vitro* glucose and insulin challenge increases Nox2 and ROS levels in coronary microvascular endothelial cells, promoting cellular senescence along with elevation of p53 (118). Furthermore, hyperlipidemia associated with aging enhances mitochondrial oxidative stress and induces plaque instability in ApoE^−/−^ mice (119). It was previously reported that Akt, which acts downstream of insulin signaling, negatively regulates the lifespan of human endothelial cells via p53/p21 signaling (120). Dietary intake of rapamycin (an inhibitor of mTOR, a molecule that acts downstream of the insulin signaling pathway) was shown to reverse age-related vascular dysfunction and oxidative stress, in association with reduced arterial expression of the senescence marker p19 (121). In the LEADER trial, a glucagon-like peptide 1 analogue (liraglutide) reduced the death rate from cardiovascular disease in patients with type 2 diabetes, as well as decreasing nonfatal myocardial infarction and nonfatal stroke (122). Another glucagon-like peptide 1 analogue (exenatide) showed beneficial vascular effects, partly via enhancing adiponectin production, and suppressed oxidative stress and inflammation in the vascular plaques of ApoE^−/−^ mice (123). Taken together, these studies indicate that, in addition to use for the inhibition of systemic metabolic disorders, suppression of cellular senescence and/or elimination of senescent cells could become next generation therapies for cardiovascular disorders.

### Conclusion and Future Directions

This review outlined the pathological role of vascular senescence in cardiovascular disease and metabolic disease. Both capillaries and arteries are critically important for delivery of nutrients and oxygen to the organs/tissues for maintenance of physiological function. Vascular endothelial cells and VSMCs have a crucial role in vascular homeostasis. Senescence of vascular cells promotes the development of age-related disorders, including heart failure, diabetes, and atherosclerotic diseases, while suppression of vascular cell senescence ameliorates phenotypic features of aging in various models. Recent findings have indicated that specific depletion of senescent cells reverses age-related changes. Considering that suppression of cellular senescence is associated with a risk of tumorigenesis, specific depletion of senescent cells may be a more promising approach to the treatment of age-related diseases. An issue that remains to be explored is the potential side effects of such treatment. For example, Demaria and colleagues found that genetic removal of senescent cells delayed wound healing in mice (124). We also need to identify the best senolytic agents, and optimize the dosage and administration and combinations for treatment of various conditions. Potential gender differences are another important research topic. Although the biological networks contributing to maintenance of homeostasis are extremely complex, it seems reasonable to explore senolytic agents that can act on specific cellular components or tissues. Several clinical trials of senolytic agents are currently ongoing. Survivors of hematopoietic stem cell transplantation are prone to premature aging, and one pilot clinical study is designed to test whether D + Q can suppress aging in these patients (Clinical Trials. Gov Identifier: NCT02652052). Another clinical trial is testing whether D + Q reduces pro-inflammatory cells obtained by skin biopsy in patients with idiopathic pulmonary fibrosis (Clinical Trials. Gov Identifier: NCT02874989). Furthermore, a clinical trial is ongoing to determine whether D + Q can reduce the senescent cell burden and frailty in patients with chronic kidney disease, as well as improving the function of adipose tissue-derived mesenchymal stem cells (Clinical Trials. Gov Identifier: NCT02848131). So far, only D + Q has been assessed in the clinical setting, and none of the current clinical trials are testing whether senolytic agents can inhibit cardiovascular disorders. However, depletion of senescent cells was demonstrated to suppress pathological progression of atherosclerotic plaque in rodents, suggesting that senolytic agents could become a next generation therapy for cardiovascular disorders (Table 1).

**Table 1:** Senolytic agents and their molecular targets

	Senolytic agents	Target	Effective type of senescent cells	Effective for aging mouse model	Effective for CVD mouse model	Ongoing clinical trials	Reference
1	Dasatinib	Dependence receptor/Src kinase/Tyrosine kinase	Irradiated human preadipocytes, human lung fibroblasts (IMR-90), MEFs, mouse preadipocytes (adipose-derived stem cells)	Yes [irradiation, progeria (*Ercc1^−/Δ^*), chronological aging]	Yes [atherosclerosis (ApoE^−/−^)]	(D + Q); chronic kidney disease, idiopathic pulmonary fibrosis, hematopoietic stem cell transplantation	(105, 107, 125–129)
2	Quercetin	Bcl-2 family, p53/p21/serpine, PI3K/AKT signaling	Irradiated HUVECs, human lung fibroblasts (IMR-90), mouse bone marrow-derived stem cells, MEFs	Yes [irradiation, progeria (*Ercc1^−/Δ^*), chronological aging]	Yes [atherosclerosis (ApoE^−/−^)]	(D + Q); chronic kidney disease, idiopathic pulmonary fibrosis, hematopoietic stem cell transplantation	(105, 107, 125–129)
3	Navitoclax (ABT-263)	Bcl-2 family (Bcl-2, Bcl-xl, and Bcl-w)	Human lung fibroblasts (IMR-90, WI-37; irradiated, RAS induction, replicative), irradiated human renal epithelial cells, MEFs	Yes (irradiation, chronological aging)	N/A	N/A	(34, 126)
4	TW-37	Bcl-2 family (Bcl-2, Bcl-xl, and Mcl-1)	MEFs passaged in high oxygen condition (20%)	N/A	N/A	N/A	(126)
5	ABT-737	Bcl-W and Bcl-XL	Human lung fibroblasts (IMR-90)	Yes (irradiation)	N/A	N/A	(125)
6	Fisetin	PI3K/AKT signaling	Irradiated HUVECs	N/A	N/A	N/A	(130)
7	A1331852	Bcl-2 family (Bcl-XL)	Irradiated human lung fibroblasts (IMR-90), HUVECs	N/A	N/A	N/A	(130)
8	A1155463	Bcl-2 family (Bcl-XL)	Irradiated human lung fibroblasts (IMR-90), HUVECs	N/A	N/A	N/A	(130)
9	Foxo4-DRI	Foxo4-p53 interaction	Irradiated human lung fibroblasts (IMR-90, WI-38), foreskin fibroblasts (BJ cells)	Yes [doxorubicin, progeria (*Xpd^TTD/TTD^*), chronological aging]	N/A	N/A	(106)
10	Alvespimycin (17-DMAG)	HSP90	*Ercc1^−/−^* MEFs	Yes [progeria (*Ercc1^−/Δ^*)]	N/A	N/A	(131)
11	Tanespimycin (17-AAG)	HSP90	*Ercc1^−/−^* MEFs	N/A	N/A	N/A	(131)
12	Geldanamycin	HSP90	*Ercc1^−/−^* MEFs	N/A	N/A	N/A	(131)
13	Piperlongumine	p53/p21 & Bcl-2 family (PUMA)	Irradiated human lung fibroblasts (WI-38)	N/A	N/A	N/A	(132)
14	Panobinostat	Histone deacetylase	Chemotherapy-induced senescent NSCLC and HNSCC cells	N/A	N/A	N/A	(133)

MEF: mouse embryonic fibroblast, HUVEC: human umbilical vein endothelial cell, NSCLC: non-small cell lung carcinoma, HNSCC: head and neck squamous cell carcinoma, D + Q: combination of dasatinib and quercetin, CVD: cardiovascular disorder, N/A: not applicable.

## Author Contributions

GK, IS, YY, TM wrote the manuscript.

## Conflict of Interest Statement

The authors declare that the research was conducted in the absence of any commercial or financial relationships that could be construed as a potential conflict of interest.
